# The Index of Microcirculatory Resistance after Primary Percutaneous Coronary Intervention Predicts Long-Term Clinical Outcomes in Patients with ST-Segment Elevation Myocardial Infarction

**DOI:** 10.3390/jcm10204752

**Published:** 2021-10-16

**Authors:** Gwang-Seok Yoon, Sung Gyun Ahn, Seong-Ill Woo, Myeong Ho Yoon, Man-Jong Lee, Seong Huan Choi, Ji-Yeon Seo, Sung Woo Kwon, Sang-Don Park, Kyoung-Woo Seo

**Affiliations:** 1 Division of Cardiology, Department of Internal Medicine, Inha University College of Medicine, Incheon 22332, Korea; shiningstonegs@gmail.com (G.-S.Y.); cardiology_icu@inha.ac.kr (M.-J.L.); seonghuan2@hanmail.net (S.H.C.); kwonsw@inha.ac.kr (S.W.K.); denki1@inha.ac.kr (S.-D.P.); 2 Division of Cardiology, Department of Internal Medicine, Yonsei University Wonju College of Medicine, Wonju 26426, Korea; sgahn@yonsei.ac.kr; 3 Department of Cardiology, Ajou University School of Medicine, Suwon 16499, Korea; jiyseo@hanmail.net (J.-Y.S.); woola@hanmail.net (K.-W.S.)

**Keywords:** index of microcirculatory resistance, ST-segment myocardial infarction, primary percutaneous coronary intervention, clinical outcome

## Abstract

The index of microcirculatory resistance (IMR) is a simple method that can measure microvascular function after primary percutaneous coronary intervention (PCI) in patients with ST-segment Elevation Myocardial Infarction (STEMI). This study is to find out whether IMR predicts clinical long-term outcomes in STEMI patients. A total of 316 patients with STEMI who underwent primary PCI from 2005 to 2015 were enrolled. The IMR was measured using pressure sensor/thermistor-tipped guidewire after primary PCI. The primary endpoint was the rate of death or hospitalization for heart failure (HF) over a mean follow-up period of 65 months. The mean corrected IMR was 29.4 ± 20.0. Patients with an IMR > 29 had a higher rate of the primary endpoint compared to patients with an IMR ≤ 29 (10.3% vs. 2.1%, *p* = 0.001). During the follow-up period, 13 patients (4.1%) died and 6 patients (1.9%) were hospitalized for HF. An IMR > 29 was associated with an increased risk of death or hospitalization for HF (OR 5.378, *p* = 0.004). On multivariable analysis, IMR > 29 (OR 3.962, *p* = 0.022) remained an independent predictor of death or hospitalization for HF with age (OR 1.048, *p* = 0.049) and symptom-to-balloon time (OR 1.002, *p* = 0.049). High IMR was an independent predictor for poor long-term clinical outcomes in STEMI patients after primary PCI.

## 1. Introduction

In ST-segment elevation myocardial infarction (STEMI), microvascular dysfunction commonly occurs even after successful revascularization of the infarct-related artery [1,2]. The presence of microvascular dysfunction after reperfusion treatment is correlated with worse clinical outcomes in patients with STEMI [3,4,5,6]. Over the past several decades, there have been many studies using both invasive and non-invasive techniques for assessment of coronary microcirculation in various clinical settings. However, despite its prognostic importance, precise assessment of microvascular dysfunction is difficult, especially in the acute phase of STEMI patients. The index of microcirculatory resistance (IMR) has been accepted as a simple and readily available method of coronary microcirculation assessment using a pressure sensor/thermistor-tipped guidewire in the catheterization laboratory immediately after primary percutaneous coronary intervention (PCI). IMR is characterized by its convenience, reproducibility, specificity in microvascular function rather than macrovascular function, and independence from hemodynamic conditions [7,8]. The value of measurement of IMR in STEMI has been evaluated in multiple studies. IMR measurement during primary PCI shows that IMR predicts myocardial infarct size, myocardial viability, myocardial salvage, and myocardial infarct characteristics [9,10,11]. Although there are several studies showing the clinical value of IMR, large-scale study of the long-term prognostic value of IMR is still lacking. The aim of this study is to determine whether IMR measured at the time of primary PCI predicts long-term clinical outcomes in large-cohort STEMI patients.

## 2. Materials and Methods

### 2.1. Study Population

This was a retrospective analysis of consecutive patients with clinical diagnosis of STEMI who underwent primary PCI at 3 centers between September 2005 and May 2015. This study enrolled STEMI patients who were relatively stable, without signs of hemodynamic or electric instability. We excluded patients with unprotected left main disease, high-degree atrioventricular block, cardiogenic shock, contraindication for the use of adenosine and a history of previous myocardial infarction at culprit vessel. Post-PCI Thrombolysis In Myocardial Infarction (TIMI) flow grades 0 or 1 were also excluded in this study. STEMI was defined as the characteristic symptom of myocardial ischemia with persistent electrocardiographic changes of ST-elevation and positive cardiac enzymes. Treatment of STEMI was performed in line with current international guidelines [12]. Aspiration thrombectomy, direct stenting, and drug injections were performed according to clinical judgement.

### 2.2. Coronary Physiologic Parameter

Coronary physiologic measurements were obtained at the culprit vessel after successful PCI. A pressure sensor-temperature sensor-tipped coronary wire (Radi Medical System, Uppsala, Sweden) was used for measuring physiologic parameters. The pressure sensor was calibrated outside the body, equalized at the tip of a guiding catheter, and then advanced to the distal two-thirds of the culprit vessel. Three injections of room-temperature 3 mL saline were administered to the coronary artery and the baseline mean transit time was measured. Intravenous adenosine (140 μg/kg/min) was then infused to induce maximal hyperemia preceded by intracoronary bolus of 200 μg nitroglycerine, and 3 more saline were injected to measure the hyperemic transit time. At the same time, the aortic and distal coronary pressures were measured during hyperemia. The coronary wedge pressure was measured after 30 s of balloon occlusion within the stented segment. IMR was defined as the distal coronary pressure at maximal hyperemia multiplied by the hyperemic mean transit time. When it was not safe to measure the wedge pressure, the corrected IMR was calculated without the wedge pressure using the Yong’s method [13]. Coronary flow reserve (CFR) was calculated as dividing the baseline mean transit time by the hyperemic mean transit time. Fractional flow reserve (FFR) was calculated as the ratio of mean distal coronary pressure to mean aortic pressure at maximal hyperemia.

### 2.3. Left Ventricular Function Assessment

A transthoracic echocardiogram was obtained within 24 h after primary PCI and at 3–6 months follow-up. Left ventricular ejection fraction (LVEF) was measured from apical four-chamber and two-chamber views using the modified Simpson method. According to the recommendation of the American Society of Echocardiography, the wall motion score index (WMSI) was assessed according to a 16-segment model as follows: normal or hyperkinesia = 1, hypokinesia = 2, akinesia = 3, and dyskinesia or aneurysmatic = 4. WMSI was calculated as the sum of all scores divided by the number of segments visualized.

### 2.4. Clinical Outcomes

The primary endpoint was a composite of death or hospitalization for heart failure (HF). Hospitalization for HF was defined as hospitalization because of signs and symptoms of HF in conjunction with non-invasive imaging findings. The secondary endpoint included the individual components of the primary outcome, as well as cardiovascular death, re-PCI including target vessel revascularization and stent thrombosis, non-fatal myocardial infarction, and stroke.

### 2.5. Statistical Analysis

Categorical variables are expressed as frequencies (percentage). Differences in categorical variables between groups were assessed using Pearson‘s chi-square tests or Fisher exact tests where appropriate. Continuous data are expressed as mean ±SD (standard deviation) and compared with the two-tailed Student *t*-test. Logistic regression analysis was performed to investigate the impact of a set of variables on endpoints. Univariable analysis was initially performed, and all the variables that exhibited a *p* < 0.05 were entered in the multivariable model, along with other established risk factors for endpoints. Event-free survival curves for primary endpoints were constructed by the Kaplan-Meier method, and statistical differences between curves were assessed by log-rank test. A *p* value < 0.05 was considered statistically significant. Statistical comparisons were performed using SPSS version 21.0 (SPSS Inc., Chicago, IL, IL, USA).

## 3. Results

### 3.1. Baseline Characteristics

A total of 316 STEMI patients who underwent primary PCI were enrolled in this study. Baseline characteristics are listed in Table 1. The mean age of the patients was 56.3 ± 11.1 years and 87.9% were male. The mean corrected IMR was 29.4 ± 20.0 (Table 2). The median and interquartile range of corrected IMR was 23.0 and 14.7–38.9, respectively (Table 2). The mean follow-up period was 65 months. Patients were divided into two groups according to the mean corrected IMR value. 126 patients (39.9%) had an IMR > 29. There were no significant differences in most baseline clinical characteristics between the two groups except that high IMR patients were older and had lower prevalence of dyslipidemia. Patients with high IMR showed significantly higher peak cardiac biomarkers. Symptom to balloon time and especially symptom to door time were significantly longer in patients with high IMR compared with those with low IMR. In echocardiographic findings at index admission, high IMR group showed lower LVEF and higher WMSI.

### 3.2. Angiographic Characteristics

In angiographic findings (Table 3), single-vessel disease was the most common (64%), and most of the infarct-related artery was left anterior descending artery (LAD). The distribution of culprit artery did not show any significant differences between the two groups. However, high IMR patients had a higher percentage of baseline TIMI flow grade 0/1 (low IMR 58.6% vs. high IMR 81.1%, *p* < 0.001) and higher rate of final TIMI myocardial perfusion (TMP) grade 0/1 (low IMR 1.2% vs. high IMR 25.0%, *p* < 0.001) than those with low IMR (Table 3).

### 3.3. Relationship between IMR and Echocardiographic Indices

**Figure 1:**
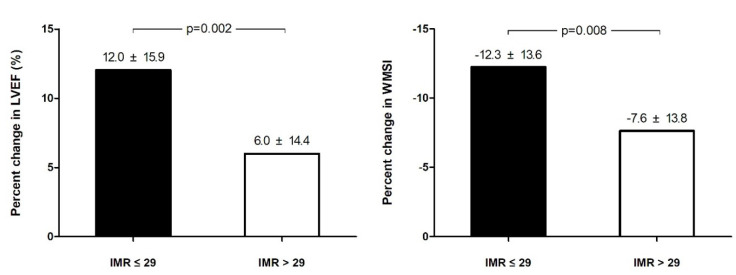
Absolute changes of echocardiographic measurements. LVEF: left ventricular ejection fraction; IMR: index of the microcirculatory resistance.

At baseline, LVEF and WMSI were statistically different between the 2 groups. At follow-up, LVEF was increased by 5.5 ± 7.0% in the low IMR group, while 2.6 ± 6.6% in the high IMR group, which equals to 11% and 5% increase in percentage change in the low IMR group and the high IMR group, respectively. The differences in the changes of LVEF were significantly higher in the low IMR group (*p* = 0.001) (Figure 1). Similar results were shown in WMSI (Figure 1).

### 3.4. Clinical Outcomes

**Figure 2:**
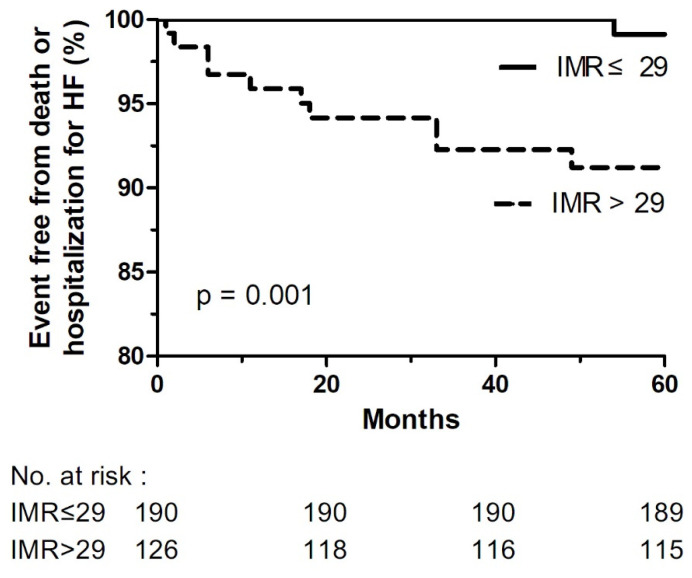
Kaplan-Meier curve of IMR. HF: heart failure; IMR: index of microcirculatory resistance.

Cumulative events during a mean follow-up period of 65 months are shown in Table 4. There were 13 (4.1%) deaths and 6 (1.9%) hospitalizations for HF in total throughout the follow-up period. High IMR patients had significantly higher rates of death or hospitalization for HF than low IMR patients (2.1% vs. 10.3%, *p* = 0.001). Similarly, all-cause mortality (2.1% vs. 7.1%, *p* = 0.028), cardiovascular death (0% vs. 4.0%, *p* = 0.006), hospitalization for HF (0% vs. 4.8%, *p* = 0.002) were significantly higher in high IMR group (Table 4). The rates of re-PCI, non-fatal myocardial infarction, and stroke were similar between the two groups. Kaplan–Meier curves for the primary endpoint are displayed in Figure 2.

**Figure 3:**
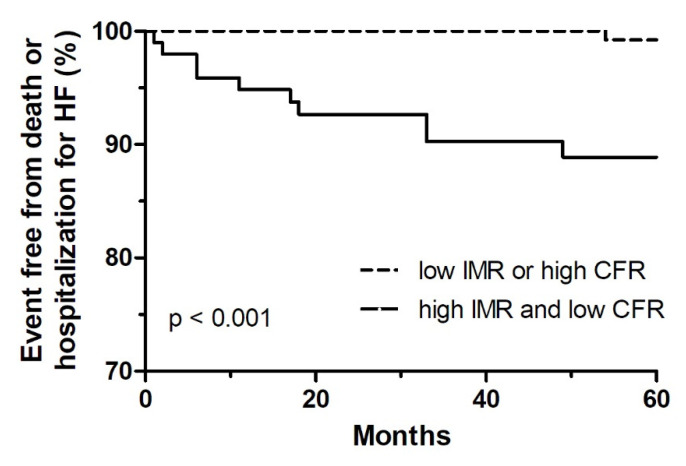
Kaplan-Meier curve of IMR & CFR. CFR: coronary flow reserve; HF: heart failure; IMR: the index of microcirculatory resistance.

The predictors of death or hospitalization for HF are shown in Table 5. Univariable logistic analysis demonstrated that age (OR: 1.071, 95% CI: 1.024–1.120, *p* = 0.003), hypertension (OR: 3.840, 95% CI: 1.319–11.184, *p* = 0.014), symptom-to-balloon time (OR: 1.002, 95% CI: 1.001–1.003, *p* = 0.006) and high IMR (OR: 5.378, 95% CI: 1.712–16.896, *p* = 0.004) were related to the primary endpoint. Two multivariable models were conducted in multivariable analysis where one used IMR alone (model A) and the other included combination of IMR and CFR variable (model B). In the model with IMR alone (model A), high IMR (OR: 3.962, 95% CI: 1.217–12.904, *p* = 0.022) remained an independent predictor of death or hospitalization for HF with age and symptom-to-balloon time (Table 5). In the model with the combination of IMR and CFR (model B), high IMR and low CFR (OR: 6.003, 95% CI: 1.831–19.678, *p* = 0.003) was also an independent predictor of death or hospitalization for HF along with age and hypertension (Table 5). The rate of death or hospitalization for HF was 10.9% in patients with IMR > 29 and CFR < 2, whereas 0.5% in patients who did not fit this range for IMR and CFR (Figure 3).

## 4. Discussion

The major finding of this study is that IMR has long-term prognostic value in STEMI patients who underwent primary PCI. Our study in a large cohort of STEMI patients showed that an IMR > 29 is associated with higher risk of death or hospitalization for HF compared with IMR ≤ 29. IMR remained an independent predictor for long-term clinical outcomes in an extended follow-up period. Compared with low IMR patients, high IMR patients had a 4-fold increase in death or hospitalization for HF. Furthermore, the combination of high IMR and low CFR values showed significant association with higher risk in clinical outcomes.

IMR has been widely used as a surrogate to invasively assess the microcirculation in STEMI patients [14]. Multiple studies have demonstrated the value of IMR measured at the time of primary PCI for STEMI patients. High IMR have been associated with larger infarct size by cardiac enzymes, less wall motion recovery at follow-up by echocardiography, and less viability by positron emission tomography [9,10]. Several studies have demonstrated correlation between IMR and CMR findings. Patients with high IMR were more likely to have microvascular obstruction (MVO), larger infarct size, less myocardial salvage, and worse LV function at follow-up [11,15,16,17,18]. However, prior studies had small sample sizes with short follow-up period and, in particular, lacked close association with hard endpoints.

In a multicenter study of 253 patients with STEMI, Fearon et al. showed that IMR > 40 measured immediately after primary PCI had a higher composite outcome of death or rehospitalization for HF during the mean follow-up period of 2.8 years (about 34 months). IMR > 40 used in the previous study had 20% of deaths or hospitalization for HF, but in contrast, this study had 10%. Mortality rate for patients with IMR > 40 was 8.8%, while it was 7.1% in this study. Moreover, they found that FFR, as well as IMR, were independent predictors of death or rehospitalization for HF [19]. Carrick et al. expanded this study to 283 patients with STEMI measured immediately after primary PCI. The IMR was an independent predictor of death or HF during a median follow-up of 845 days (about 28 months), but the combination of IMR > 40 and CFR ≤ 2 did not have additional prognostic value compared with an IMR > 40 [20].

With larger sample size (*n* = 316) and longer mean follow-up period (65 months), the present study found similar results with previous studies. In our study, patients with IMR above 29 of the mean value had significantly higher rates of death or hospitalization for HF than low IMR patients (10.3% vs. 2.1%, *p* = 0.001), and all-cause mortality (7.1% vs. 2.1%, *p* = 0.028), cardiovascular death rate (4.0% vs. 0%, *p* = 0.006), and hospitalization for HF rate (4.8% vs. 0%, *p* = 0.002). In addition, the LVEF and WMSI at follow-up were significantly worse in the IMR > 29.

Our data showed that high IMR is associated with higher cardiac biomarkers, lower LVEF, higher WMSI, and longer symptom-to-balloon time. Taking all of these into account, high correlation between IMR and the degree of myocardial damage could partially explain the relationship between high IMR and poor clinical outcomes. In our study, occurrence of coronary artery events between the two groups was not statistically significant, including non-fatal myocardial infarction and re-PCI. However, it is noteworthy that high IMR patients were highly likely to result in death or hospitalization for HF. These findings all point out that high IMR is significantly correlated with microvascular dysfunction, which leads to long-term adverse clinical outcomes in patients with STEMI.

Compared with the study of Fearon et al., our study included larger populations and had a much longer follow-up period. In addition, FFR measured in our study was not predictive to long-term clinical outcomes; only 4.1% had a FFR < 0.8 after primary PCI. Despite many studies suggesting different IMR cutoff values, IMR > 40 has been widely accepted until now to have prognostic value to clinical outcomes [11,18,19]. However, the threshold of 40 needs further validation as the cutoff value. In a study of 1096 patients with ischemic heart disease, Lee et al. demonstrated that determinants of high IMR were previous myocardial infarction, right coronary artery (RCA), female, and obesity [21]. A higher IMR value of the RCA could be related to longer length of the vessel leading to a slightly longer mean transit time or to a smaller amount of myocardial mass, which can influence resistance [22]. When comparing to studies with higher mean IMR values, infarct artery distribution accounted for 44–55% of LAD and 36–44% of RCA, whereas 78% and 16% in our study, respectively [17,19,23]. Indeed, the mean IMR value was not much different in other studies showing a culprit artery distribution similar to our study [10,16,24]. Giovanni et al. found that when a threshold of 40 is adopted for IMR, there is discordance between IMR and MVO in one-third of the cases [25]. On the basis of recent studies evaluating IMR, the normal range of IMR is considered to be <25 U [26,27]. The IMR > 29 can be a sufficient cutoff value for predicting clinical outcomes. However, we should be careful when interpreting cutoff value of IMR by considering various factors.

When comparing with the study of Carrick et al., which further extended the study of Fearon et al., combination of IMR and CFR was a stronger predictor in clinical outcomes compared to IMR as single parameter (IMR > 29 OR 3.962 vs. IMR > 29 & CFR < 2 OR 6.003). In previous studies, the combination of IMR and CFR had an independent predictive value for MVO detection and showed association with myocardial viability and clinical outcomes. Ahn et al. assessed the usefulness of combination of IMR and CFR values for predicting microvascular obstruction by cardiac magnetic resonance imaging in patients with primary PCI for STEMI. The combination of high IMR and low CFR was highly predictive of microvascular obstruction [14]. Park et al. studied 89 STEMI patients who underwent primary PCI and found that IMR and CFR measured immediately after PCI were correlated with cardiovascular and cerebrovascular events [24]. These findings are in line with our research, which also found that high IMR and low CFR resulted in worse clinical outcomes in STEMI patients. A high IMR and low CFR lead to microvascular dysfunction, whereas low IMR and high CFR indicate normal microcirculatory integrity. But there are cases when the paired values are both high or low [15]. A total of 41% of patients in our study showed such discordance in paired values, whereas 35–62% showed such discordance in other studies [15,24,28]. CFR reflects the epicardial and microvascular vasodilatory capacity, whereas IMR does not reflect vasodilatory reserve but only reflects microvascular resistance. In addition, CFR is much dependent on hemodynamics, which means that it has greater variability [29]. Furthermore, as we excluded hemodynamically unstable patients, we can say that high risk patients were not included in this study. Therefore, discordant paired values of IMR and CFR can be observed. We found that the combination of IMR and CFR may more precisely predict long-term clinical outcomes of death or hospitalization for HF.

Major limitations of the present study were that it was observational and retrospective. Also, the number of events in this study was relatively small. Coronary measurements were not undertaken in patients with any clinical concerns. Hence, the study could have included largely relatively low risk patients.

## 5. Conclusions

IMR measured after primary PCI can serve as a strong predictor of long-term outcomes in STEMI patients. Compared with low IMR in STEMI patients, high IMR had a significant correlation with the development of adverse events including death alone and death or hospitalization for HF.

## Figures and Tables

**Table 1:** Baseline clinical characteristics, laboratory findings, and echocardiographic parameters.

	Total	IMR ≤ 29 U	IMR > 29 U	
		(*n* = 190)	(*n* = 126)	
Age, years	56.3 ± 11.1	54.9 ± 10.6	58.3 ± 11.7	0.008
Male, *n* (%)	277 (87.9)	171 (90.5)	106 (84.1)	0.090
BMI, kg/m^2^	24.6 ± 3.1	24.5 ± 2.8	24.6 ± 3.5	0.805
Comorbidities, *n* (%)				
Hypertension	127 (40.2)	71 (37.4)	56 (44.4)	0.209
Diabetes	83 (26.3)	46 (24.2)	37 (29.4)	0.308
Dyslipidemia	139 (44.0)	102 (53.7)	37 (29.4)	0.000
Prior PCI	5(2.4)	2(1.7)	3(3.3)	0.451
Smoking	238 (75.3)	145 (76.3)	93 (73.8)	0.613
Systolic blood pressure, mmHg	131.6 ± 23.5	131.1 ± 21.9	132.6 ± 26.2	0.614
Diastolic blood pressure, mmHg	81.7 ± 15.7	81.7 ± 14.3	81.6 ± 17.9	0.941
Heart rate, bpm	77.2 ± 15.4	77.9 ± 15.4	75.8 ± 15.4	0.292
Symptom-to-balloon time, min	276.1 ± 232.2	254.1 ± 225.6	308.2 ± 238.8	0.046
Symptom-to-door time, min	201.1 ± 212.4	170.6 ± 173.0	241.5 ± 250.7	0.025
Door-to-balloon time, min	80.3 ± 82.0	79.8 ± 87.1	81.0 ± 74.0	0.903
Medications at discharge, *n* (%)				
Aspirin	316 (100)	190 (100)	126 (100)	N/A
Clopidogrel	263 (83.2)	157 (82.6)	106 (84.1)	0.728
Ticagrelor	46 (31.5)	29 (32.6)	17 (29.8)	0.726
Prasugrel	5 (2.4)	4 (3.4)	1 (1.1)	0.284
ARB or ACEi	155 (91.2)	92 (91.1)	63 (91.3)	0.961
β-Blocker	160 (94.1)	98 (97.0)	62 (89.9)	0.051
Statin	166 (97.6)	100 (99.0)	66 (95.7)	0.156
Laboratory values				
WBC, ×10^9^/L	11.7 ± 3.8	11.7 ± 3.6	11.7 ± 3.9	0.888
Hb, g/dL	14.7 ± 1.8	14.8 ± 1.7	14.6 ± 1.9	0.350
Plt, ×10^9^/L	252.9 ± 69.0	252.4 ± 68.5	253.6 ± 70.1	0.874
NT-proBNP, pg/mL	741.0 ± 3235.9	445.5 ± 2099.7	1223.1 ± 4512.5	0.245
CRP, mg/dL	0.52 ± 1.74	0.44 ± 0.92	0.65 ± 2.59	0.503
Glucose, mg/dL	163.0 ± 58.5	156.8 ± 52.8	172.4 ± 65.4	0.028
HbA1c, %	6.5 ± 1.4	6.4 ± 1.2	6.6 ± 1.6	0.374
BUN, mg/dL	15.6 ± 6.4	16.0 ± 6.3	15.5 ± 6.6	0.935
Creatinine, mg/dL	1.01 ± 0.28	1.02 ± 0.25	1.00 ± 0.32	0.515
AST, IU/L	59.3 ± 87.3	49.0 ± 64.2	77.6 ± 115.8	0.031
ALT, IU/L	37.3 ± 29.5	33.9 ± 20.1	43.4 ± 40.5	0.037
Total cholesterol, mg/dL	186.3 ± 40.9	185.1 ± 37.7	188.0 ± 45.5	0.541
TG, mg/dL	130.6 ± 98.9	130.7 ± 94.1	130.4 ± 106.1	0.978
HDL, mg/dL	43.4 ± 10.0	43.1 ± 9.9	43.7 ± 10.1	0.657
LDL, mg/dL	117.2 ± 37.3	116.4 ± 34.8	118.2 ± 40.9	0.677
Peak CK, IU/L	2636.1 ± 2489.3	1958.2 ± 2052.4	3842.9 ± 2741.5	0.000
Peak CK-MB, mg/mL	259.3 ± 184.8	214.7 ± 171.9	327.2 ± 183.9	0.000
Peak Trop-I, ng/mL	63.2 ± 69.5	56.3 ± 61.3	74.0 ± 79.6	0.046
Echocardiographic measure				
End-diastolic dimension, mm	50.2 ± 4.9	50.0 ± 4.7	50.5 ± 5.2	0.322
End-systolic dimension, mm	36.4 ± 5.4	36.1 ± 5.2	37.0 ± 5.8	0.157
Left atrial dimension, mm	38.1 ± 3.7	38.2 ± 3.6	38.1 ± 3.9	0.849
Left ventricle mass index, g/m^2^	105.3 ± 21.6	102.8 ± 17.2	109.2 ± 26.9	0.115
E/E′ ratio	10.9 ± 3.3	10.2 ± 2.9	11.8 ± 3.6	0.001
Ejection fraction, %	47.3 ± 7.6	48.4 ± 7.8	45.6 ± 6.9	0.001
Wall motion score index	1.55 ± 0.29	1.50 ± 0.29	1.64 ± 0.27	0.000
Values are mean ± SD or *n* (%).				

**Table 2:** Post-procedural coronary physiology measurements.

	Total	IMR ≤ 29 U	IMR > 29 U	*p*
		(*n* = 190)	(*n* = 126)	
Pa hyperemia, mmHg	88.8 ± 15.5	88.3 ± 14.3	89.7 ± 17.2	0.451
Pd hyperemia, mmHg	82.0 ± 15.5	80.7 ± 14.2	84.1 ± 17.0	0.056
Tmn rest, sec	0.60 ± 0.36	0.45 ± 0.26	0.84 ± 0.37	0.000
Tmn hyperemia, sec	0.36 ± 0.25	0.22 ± 0.07	0.59 ± 0.26	0.000
FFR	0.92 ± 0.07	0.91 ± 0.07	0.93 ± 0.07	0.063
CFR	1.94 ± 1.29	2.18 ± 1.47	1.56 ± 0.80	0.000
IMR	27.7 ± 17.6	16.7 ± 5.1	44.4 ± 16.6	0.000
IMRc	29.4 ± 20.0	17.1 ± 5.2	48.1 ± 19.5	0.000

Values are mean ±SD. Pa: aortic pressure; Pd: distal pressure; Tmn: mean transit time; CFR: coronary flow reserve; FFR: fractional flow reserve; IMR: index of microcirculatory resistance; IMRc: corrected IMR.

**Table 3:** Angiographic characteristics.

	Total	IMR ≤ 29 U	IMR > 29 U	*p*
		(*n* = 190)	(*n* = 126)	
Baseline characteristics				
culprit artery, *n* (%)				0.338
LAD	248 (78.2)	151 (79.1)	97 (77.0)	
LCX	20 (6.3)	9 (4.7)	11 (8.7)	
RCA	49 (15.5)	31 (16.2)	18 (14.3)	
number of vessel, *n* (%)				0.111
1	202 (64.1)	130 (68.4)	72 (57.6)	
2	86 (27.3)	44 (23.2)	42 (33.6)	
3	27 (8.6)	16 (8.4)	11 (8.8)	
≥2	113 (35.9)	62 (32.6)	54 (40.8)	0.139
TIMI grade before PCI, *n* (%)				0.000
0/1	201 (67.9)	102 (58.6)	99 (81.1)	0.000
2	65 (22.0)	46 (26.4)	19 (15.6)	0.026
3	30 (10.1)	26 (14.9)	4 (3.3)	0.001
Post-procedural characteristics				
Stent diameter, mm	3.20 ± 0.36	3.19 ± 0.36	3.21 ± 0.38	0.502
Stent length, mm	25.9 ± 9.5	25.7 ± 9.7	26.2 ± 9.1	0.622
TIMI grade after PCI, *n* (%)				0.000
0/1	0	0	0	N/A
2	37 (13.3)	6 (3.8)	31 (26.1)	0.000
3	242 (86.7)	154 (96.3)	88 (73.9)	0.000
TMP grade after PCI, *n* (%)				0.000
0/1	32 (11.2)	2 (1.2)	30 (25.0)	
2	119 (41.8)	68 (41.2)	51 (42.5)	0.828
3	134 (47.0)	95 (57.6)	39 (32.5)	0.000

Values are mean ±SD or *n* (%). LAD: left anterior descending artery; LCX: left circumflex artery; RCA: right coronary artery; TIMI: thrombolysis in myocardial infarction; PCI: percutaneous coronary intervention; TMP: TIMI myocardial perfusion.

**Table 4:** Long-term clinical outcomes.

		**IMR**		***p*-Value**
	**Total (*n* = 316)**	**IMR ≤ 29 (*n* = 190)**	**IMR > 29 (*n* = 126)**	
Primary endpoint				
Death or hospitalization for HF	17 (5.4)	4 (2.1)	13 (10.3)	0.001
Secondary endpoint				
All-death	13 (4.1)	4 (2.1)	9 (7.1)	0.028
Cardiovascular death	5 (1.6)	0 (0)	5 (4.0)	0.006
Hospitalization for HF	6 (1.9)	0 (0)	6 (4.8)	0.002
Re-PCI	24 (7.6)	14 (7.4)	10 (7.9)	0.862
TLR	8 (2.5)	4 (2.5)	4 (4.3)	0.425
ST	2 (0.6)	1 (1.1)	1 (1.8)	0.749
Non-fatal MI	6 (1.9)	2 (1.1)	4 (3.2)	0.178
Stroke	8 (2.5)	5 (3.1)	3 (3.2)	0.958
		**IMR & CFR**		***p*-Value**
	**Total (*n* = 314)**	**IMR ≤ 29 or CFR ≥ 2 (*n* = 213)**	**IMR > 29 and CFR < 2 (*n* = 101)**	
Primary endpoint				
Death or hospitalization for HF	17 (5.4)	4 (1.9)	13 (12.9)	<0.001
Secondary endpoint				
All-death	13 (4.1)	4 (1.9)	9 (8.9)	0.006
Cardiovascular death	5 (1.6)	0 (0)	5 (5.0)	0.003
Hospitalization for HF	6 (1.9)	0 (0)	6 (5.9)	0.001
Re-PCI	24 (7.6)	16 (7.6)	8 (7.9)	1.000
TLR	8 (2.5)	5 (2.8)	3 (3.9)	0.699
ST	2 (0.6)	1 (1.0)	1 (2.1)	0.542
Non-fatal MI	6 (1.9)	2 (0.9)	4 (4.0)	0.089
Stroke	8 (2.5)	5 (2.8)	3 (3.9)	0.699

Values are *n* (%). HF: heart failure; PCI: percutaneous coronary intervention; TLR: target lesion revascularization; ST: stent thrombosis; MI: myocardial infarction.

**Table 5:** Logistic regression analysis for independent predictors of death or hospitalization for HF.

	Univariate Analysis	*p*-Value	Multivariable Analysis (Model A)	*p*-Value	Multivariable Analysis (Model B)	*p*-Value
	OR (95% CI)		OR (95% CI)		OR (95% CI)	
Age	1.071 (1.024 to 1.120)	0.003	1.048 (1.000 to 1.098)	0.049	1.050 (1.002 to 1.100)	0.040
Female	1.610 (0.441 to 5.884)	0.471				
Hypertension	3.840 (1.319 to 11.184)	0.014	3.056 (0.983 to 9.504)	0.054	3.284 (1.045 to 10.323)	0.042
Diabetes	2.655 (0.989 to 7.128)	0.053				
Smoking	0.445 (0.163 to 1.121)	0.113				
Post-PCI TMP grade <3	1.287 (0.476 to 3.481)	0.620				
IMR > 29	5.378 (1.712 to 16.896)	0.004	3.962 (1.217 to 12.904)	0.022		
CFR < 2	3.923 (0.880 to 17.489)	0.073				
IMR > 29 & CFR < 2	7.719 (2.449 to 24.328)	0.000			6.003 (1.831 to 19.678)	0.003
Symptom-to-balloon time	1.002 (1.001 to 1.003)	0.006	1.002 (1.000 to 1.003)	0.049	1.002 (1.000 to 1.003)	0.057
Culprit artery						
LAD	0.899 (0.284–2.850)	0.856				
LCX	0.924 (0.116–7.347)	0.941				
RCA	1.183 (0.327–4.281)	0.798				
LVEF < 40%	1.258 (0.583–2.715)	0.558				
Peak CK-MB	1.000 (0.997 to 1.003)	0.932				

PCI: percutaneous coronary intervention; TMP: TIMI myocardial perfusion; IMR: index of microcirculatory resistance; CFR: coronary flow reserve; LAD: left anterior descending artery; LCX: left circumflex artery; RCA: right coronary artery; LVEF: left ventricular ejection fraction.

## Data Availability

The data presented in this study are available upon request from the corresponding author. The data are not publicly available, because of restrictions such as privacy or ethical issues.

## References

[1] Weir R.A.P., Murphy C.A., Petrie C.J., Martin T.N., Balmain S., Clements S., Steedman T., Wagner G.S., Dargie H.J., McMurray J.J.V. (2010). Microvascular obstruction remains a portent of adverse remodeling in optimally treated patients with left ventricular systolic dysfunction after acute myocardial infarction. Circ. Cardiovasc. Imaging.

[2] Hamirani Y.S., Wong A., Kramer C.M., Salerno M. (2014). Effect of microvascular obstruction and intramyocardial hemorrhage by CMR on LV remodeling and outcomes after myocardial infarction: A systematic review and meta-analysis. JACC Cardiovasc. Imaging.

[3] Niccoli G., Burzotta F., Galiuto L., Crea F. (2009). Myocardial no-reflow in humans. J. Am. Coll. Cardiol..

[4] Gibson C.M., Cannon C.P., Murphy S.A., Ryan K.A., Mesley R., Marble S.J., McCabe C.H., Van de Werf F., Braunwald E. (2000). Relationship of TIMI myocardial perfusion grade to mortality after administration of thrombolytic drugs. Circulation.

[5] Wu K.C., Zerhouni E.A., Judd R.M., Lugo-Olivieri C.H., Barouch L.A., Schulman S.P., Blumenthal R.S., Lima J.A.C. (1998). Prognostic significance of microvascular obstruction by magnetic resonance imaging in patients with acute myocardial infarction. Circulation.

[6] Herzog B.A., Husmann L., Valenta I., Gaemperli O., Siegrist P.T., Tay F.M., Burkhard N., Wyss C.A., Kaufmann P.A. (2009). Long-term prognostic value of 13N-ammonia myocardial perfusion positron emission tomography added value of coronary flow reserve. J. Am. Coll. Cardiol..

[7] Fearon W.F., Balssm L.B., Farouque H.M.O., Robbins R.C., Fitzgerald P.J., Yock P.G., Yeung A.C. (2003). Novel index for invasively assessing the coronary microcirculation. Circulation.

[8] Ng M.K.C., Yeung A.C., Fearon W.F. (2006). Invasive assessment of the coronary microcirculation: Superior reproducibility and less hemodynamic dependence of index of microcirculatory resistance compared with coronary flow reserve. Circulation.

[9] Fearon W.F., Shah M., Martin N., Brinton T., Wilson A., Tremmel J.A., Schnittger I., Lee D.P., Vagelos R.H., Fitzgerald P.J. (2008). Predictive value of the index of microcirculatory resistance in patients with ST-segment elevation myocardial infarction. J. Am. Coll. Cardiol..

[10] Lim H.S., Yoon M.H., Tahk S.J., Yang H.M., Choi B.J., Choi S.Y., Sheen S.S., Hwang G.S., Kang S.J., Shin J.H. (2009). Usefulness of the index of microcirculatory resistance for invasively assessing myocardial viability immediately after primary angioplasty for anterior myocardial infarction. Eur. Heart J..

[11] McGeoch R., Watkins S., Berry C., Steedman T., Davie A., Byrne J., Hillis S., Lindsay M., Robb S., Dargie H. (2010). The index of microcirculatory resistance measured acutely predicts the extent and severity of myocardial infarction in patients with ST-segment elevation myocardial infarction. JACC Cardiovasc. Interv..

[12] Windecker S., Kolh P., Alfonso F., Collet J.P., Cremer J., Falk V., Filippatos G., Hamm C., Head S.J., Juni P. (2014). 2014 ESC/EACTS Guidelines on myocardial revascularization: The Task Force on Myocardial Revascularization of the European Society of Cardiology (ESC) and the European Association for Cardio-Thoracic Surgery (EACTS) Developed with the special contribution of the European Association of Percutaneous Cardiovascular Interventions (EAPCI). Eur. Heart J..

[13] Yong A.S., Layland J., Fearon W.F., Ho M., Shah M.G., Daniels D., Whitbourn R., MacIsaac A., Kritharides L., Wilson A. (2013). Calculation of the index of microcirculatory resistance without coronary wedge pressure measurement in the presence of epicardial stenosis. JACC Cardiovasc. Interv..

[14] Bulluck H., Foin N., Tan J.W., Low A.F., Sezer M., Hausenloy D.J. (2017). Invasive Assessment of the Coronary Microcirculation in Reperfused ST-Segment-Elevation Myocardial Infarction Patients: Where Do We Stand?. Circ. Cardiovasc. Interv..

[15] Ahn S.G., Hung O.Y., Lee J.W., Lee J.H., Youn Y.J., Ahn M.S., Kim J.Y., Yoo B.S., Lee S.H., Yoon J.H. (2016). Combination of the Thermodilution-Derived Index of Microcirculatory Resistance and Coronary Flow Reserve Is Highly Predictive of Microvascular Obstruction on Cardiac Magnetic Resonance Imaging after ST-Segment Elevation Myocardial Infarction. JACC Cardiovasc. Interv..

[16] Ahn S.G., Lee S.H., Lee J.H., Lee J.W., Youn Y.J., Ahn M.S., Kim J.Y., Yoo B.S., Youn J.H., Choe K.H. (2014). Efficacy of combination treatment with intracoronary abciximab and aspiration thrombectomy on myocardial perfusion in patients with ST-segment elevation myocardial infarction undergoing primary coronary stenting. Yonsei Med. J..

[17] Fukunaga M., Fujii K., Kawasaki D., Sawada H., Miki K., Tamaru H., Imanaka T., Iwasaku T., Nakata T., Shibuya M. (2014). Thermodilution-derived coronary blood flow pattern immediately after coronary intervention as a predictor of microcirculatory damage and midterm clinical outcomes in patients with ST-segment-elevation myocardial infarction. Circ. Cardiovasc. Interv..

[18] Payne A.R., Berry C., Doolin O., McEntegart M., Petrie M.C., Lindsay M., Hood S., Carrick D., Tzemos N., Weale P. (2012). Microvascular Resistance Predicts Myocardial Salvage and Infarct Characteristics in ST-Elevation Myocardial Infarction. J. Am. Heart Assoc..

[19] Fearon W.F., Low A.F., Yong A.S., McGeoch R., Berry C., Shah M.G., Ho M.Y., Kim H.S., Loh J.P., Oldroyd K.G. (2013). Prognostic value of the Index of Microcirculatory Resistance measured after primary percutaneous coronary intervention. Circulation.

[20] Carrick D., Haig C., Ahmed N., Carberry J., May V.T.Y., McEntegart M., Petrie M.C., Eteiba H., Lindsay M., Hood S. (2016). Comparative Prognostic Utility of Indexes of Microvascular Function Alone or in Combination in Patients with an Acute ST-Segment-Elevation Myocardial Infarction. Circulation.

[21] Lee J.M., Layland J., Jung J.H., Lee H.J., Echavarria-Pinto M., Watkins S., Yong A.S., Doh J.H., Nam C.W., Shin E.S. (2015). Integrated physiologic assessment of ischemic heart disease in real-world practice using index of microcirculatory resistance and fractional flow reserve: Insights from the International Index of Microcirculatory Resistance Registry. Circ. Cardiovasc. Interv..

[22] Echavarria-Pinto M., van de Hoef T.P., Nijjer S., Gonzalo N., Nombela-Franco L., Ibanez B., Sen S., Petraco R., Jimenez-Quevedo P., Nunez-Gil I.J. (2017). Influence of the amount of myocardium subtended to a coronary stenosis on the index of microcirculatory resistance. Implications for the invasive assessment of microcirculatory function in ischaemic heart disease. EuroIntervention.

[23] Cuculi F., Maria G.L.D., Meier P., Dall’Armellina E., de Caterina A.R., Channon K.M., Prendergast B.D., Choudhury R.C., Forfar J.C., Kharbanda R.K. (2014). Impact of microvascular obstruction on the assessment of coronary flow reserve, index of microcirculatory resistance, and fractional flow reserve after ST-segment elevation myocardial infarction. J. Am. Coll. Cardiol..

[24] Park S.D., Baek Y.S., Lee M.J., Kwon S.W., Shin S.H., Woo S.I., Kim D.H., Kwan J., Park K.S. (2016). Comprehensive assessment of microcirculation after primary percutaneous intervention in ST-segment elevation myocardial infarction: Insight from thermodilution-derived index of microcirculatory resistance and coronary flow reserve. Coron. Artery Dis..

[25] De Maria G.L., Alkhalil M., Wolfrum M., Fahrmi G., Borlotti A., Gaughran L., Dawkins S., Lnagrish J.P., Lucking A.J., Choudhury R.P. (2019). Index of Microcirculatory Resistance as a Tool to Characterize Microvascular Obstruction and to Predict Infarct Size Regression in Patients with STEMI Undergoing Primary PCI. JACC Cardiovasc. Imaging.

[26] Ford T.J., Ong P., Sechtem U., Beltrame J., Camici P.G., Crea F., Kaski J.C., Bairey Merz C.N., Pepine C.J., Shimokawa H. (2020). Assessment of Vascular Dysfunction in Patients Without Obstructive Coronary Artery Disease: Why, How, and When. JACC Cardiovasc. Interv..

[27] Kunadian V., Chieffo A., Camici P.G., Berry C., Escaned J., Maas A., Prescott E., Karam N., Appelman Y., Fraccaro C. (2020). An EAPCI Expert Consensus Document on Ischaemia with Non-Obstructive Coronary Arteries in Collaboration with European Society of Cardiology Working Group on Coronary Pathophysiology & Microcirculation Endorsed by Coronary Vasomotor Disorders International Study Group. Eur. Heart J..

[28] Lim H.S., Tahk S.J., Yoon M.H., Woo S.I., Choi W.J., Hwang J.W., Li D.H., Seo K.W., Park J.S., Kim J.W. (2007). A Novel Index of Microcirculatory Resistance for Invasively Assessing Myocardial Viability after Primary Angioplasty for Treating Acute Myocardial Infarction: Comparison with FDG-PET Imaging. Korean Circ. J..

[29] Barbato E., Aarnnoudse W., Aengevaeren W.R., Werner G., Klauss W., Bojara W., Herzfeld I., Oldroyd K.G., Pijls N.H.J., de Bruyne B. (2004). Validation of coronary flow reserve measurements by thermodilution in clinical practice. Eur. Heart J..

